# The Need for Psychiatric Treatment among Polish Users of Psychoactive Substances Is Increasing: This and Other Results from the Newest PolDrugs Survey

**DOI:** 10.3390/medicina59050908

**Published:** 2023-05-09

**Authors:** Gniewko Więckiewicz, Julia Marek, Iga Stokłosa, Sandra Szafoni, Szymon Pluta, Katarzyna Smukowska, Gabriela Żebrowska, Maciej Stokłosa, Piotr Gorczyca, Robert Pudlo

**Affiliations:** 1 Department of Psychiatry, Faculty of Medical Sciences in Zabrze, Medical University of Silesia, 40-055 Katowice, Poland; 2 Students’ Scientific Circle in Department of Psychiatry, Faculty of Medical Sciences in Zabrze, Medical University of Silesia, 40-055 Katowice, Poland; 3 Social Drug Policy Initiative, 02-662 Warsaw, Poland; 4 Department of Psychoprophylaxis, Faculty of Medical Sciences in Zabrze, Medical University of Silesia, 40-055 Katowice, Poland

**Keywords:** drugs, PolDrugs, psychoactive substances, psilocybin, DMT, marihuana, MDMA, psychiatry, epidemiology

## Abstract

*Background and Objectives*: PolDrugs is the largest Polish naturalistic nationwide survey to present basic demographic and epidemiological data that could potentially prevent harm from illicit substances intake in drugs users. The most recent results were presented in 2021. The goal of this year’s edition was to re-present the above data and compare it to the previous edition’s data to identify and describe the differences. *Materials and Methods*: The survey included original questions about basic demographics, substance use, and psychiatric treatment. The survey was administered via the Google Forms platform and promoted via social media. The data was collected from 1117 respondents. *Results*: People of all ages use a variety of psychoactive substances in many situations. The three most commonly used drugs are marijuana, 3,4-methylenedioxymethamphetamine, and hallucinogenic mushrooms. The most common reason for seeking professional medical help was amphetamine use. A total of 41.7 percent of respondents were receiving psychiatric treatment. The three most common psychiatric diagnoses among the respondents were depressive disorders, anxiety disorders, and ADHD. *Conclusions*: Key findings include increases in the use of psilocybin and DMT, increases in the use of heated tobacco products, and a near doubling in the percentage of individuals receiving psychiatric help in the past two years. These issues are discussed in the discussion section of this paper, which also addresses the limitations to the article.

## 1. Background and Objectives

There is no doubt that psychoactive substances have been, are and will remain present in our world. Only attitudes toward them are changing, as some that were once illegal are becoming completely legal in many countries (such as marijuana) or legal for medical use (such as 3,4-methylenedioxymethamphetamine or psilocybin) [1,2]. Given these developments, which are likely to have an impact on the popularity and use of substances outside the regulated market, epidemiological and demographic data on recreational users of psychoactive substances need to be regularly updated. One project aimed at providing such data is “PolDrugs”, a biennial project launched in 2020 by psychiatrists Gniewko Więckiewicz and Robert Pudlo to provide baseline data on Polish recreational users of psychoactive substances. The last time the project was conducted and the findings published was in 2021. The aim was to present the results to psychiatrists in order to draw their attention to the fact that, in addition to the patients of addiction treatment clinics, there is a significant group of people who use psychoactive substances and who are not patients of such health care institutions or who go to the doctor for other reasons [3]. It turned out that the project was of great interest not only to psychiatrists, as the media all over the country shared the data from the article summarizing PolDrugs 2021 [4,5]. According to the authors, this is most likely due to the fact that Poland is a relatively conservative country in terms of the laws on the possession of psychoactive substances, which highlights the need for reliable studies. Considering the fact that attitudes towards psychoactive substances are dynamically changing all over the world, it was decided that the answers to the same questions would be collected again, exactly two years after the last data collection, in order to evaluate possible changes and recent trends in consumption. This time, in order to ensure the most reliable analysis of the collected data, representatives from the Social Drug Policy Initiative, the largest non-governmental organization in the country that works to reduce harm from psychoactive substance use, were invited to co-author the manuscript. The goal of the survey was to present key demographic and substance abuse data from the perspective of a psychiatrist practicing in Poland and to compare this data with the data from the same project two years ago.

## 2. Materials and Methods

### 2.1. Survey

The survey included questions on basic demographics, exposure to psychoactive substances, and psychiatric treatment history. It was hosted on the Google Forms platform, a user-friendly tool for authors and respondents to collect or submit data via the Internet. Before starting the survey, users had to confirm that they voluntarily consented to the release and further processing of the data, and in the case of minors, that they had the consent of their legal guardian. To ensure a sense of security, the survey was completely anonymously; neither IP addresses nor e-mail addresses were collected by the authors. Since the questions were asked on the online platform, it is not possible to include the original version of the survey as an attachment to the article. Therefore, all questions and the original layout of our survey have been included in tables in the Results section without changing the content and should be considered as such. The collected anonymous data were processed in accordance with the personal data protection regulations in force in the Republic of Poland and the European Union.

### 2.2. Data Collection

The data were collected for one month, from 21 November 2022 until 22 December 2022. A total of 1117 correctly completed surveys were collected. Since the data collected were to be compared with the results from the previous edition of PolDrugs, they were collected in the same way as the last time, i.e., the data were collected through social media (Facebook, Instagram). The survey was shared on pages for psychoactive substance users and groups related to stimulant use, such as pages about music festivals, and club culture, and pages for artists, students, and pupils. Hashtags related to stimulants were also used. Activists from the Social Drug Policy Initiative and the Polish Drug Policy Network participated in the dissemination of the survey.

### 2.3. Statistical Analysis

Because the data collection method used in PolDrugs involves random sampling, which places a burden on the survey sample, and there is a possibility of bias that could distort the survey results due to low external validity, only descriptive statistical analysis was presented in the results, while inferential analysis was not carried out to avoid any misleading findings.

## 3. Results

The youngest respondent was 13 years old, and the oldest was 62 years old. The average age of the respondents was 27 years. Most of the psychoactive substance users surveyed were female (50.2%), heterosexually oriented (72.2%), and in an informal relationship (48.9%). Most users had a high school degree (45.3%), live in a large city (64.2%), and work full-time (40.6%). Those who earnt a wage were most likely to have a net income of more than PLN 5000 (31.7%). Detailed demographic data are presented in Table 1.

A total of 704 respondents use tobacco (63%), choosing classic cigarettes most often (27.8%), and 907 respondents (81.2%) use alcohol. The largest group drinks 2–3 times a month (27.4%) and chooses beer most often (50.4%). Moreover, 12.9% of respondents had considered getting help for their alcohol consumption. Detailed results on nicotine and alcohol consumption can be found in Table 2.

The average age of first contact with psychoactive substances is 17 years old. The most commonly used psychoactive substance other than alcohol, caffeine, and nicotine is marihuana, with 98.1% of respondents having used it in their lifetime and 86.2% of respondents having used it in the past 12 months. Psychoactive substances are most commonly used once every few months or less (34.9%), with friends (54.3%), and at home (47.5%). The majority of users buy psychoactive substances from friends (57.4%) and spend up to PLN 100 per month on them (41.7%). It was found that 77.5% of respondents never test their psychoactive substances with colorimetric reagents or in a laboratory, and 40.9% of respondents measure the dose by visual observation. Overall, 92.7% of respondents have tried to learn about the safety of psychoactive substances through scientific articles or non-governmental organizations (NGOs). A total of 51.7% of respondents have neglected everyday tasks at least once in their lives due to psychoactive substance use, and 9% have been in trouble with the law because of it. The details are shown in Table 3.

The vast majority of respondents had not considered seeking professional help for psychoactive substance use (82.5%), while 72 respondents (6.4%) had sought medical help shortly after using psychoactive substances, most frequently after using amphetamines (23.6%). The detailed results are presented in Table 4.

Overall, 651 respondents (58.3%) are being or have been treated by a psychiatrist in the past, most often in private practice. Most respondents had seen a physician for depressive disorders. A total of 171 individuals in this group had attempted suicide at least once. In most cases, the first visit to the doctor was preceded by the use of psychoactive substances (63.3%). Most psychiatrists asked about the psychoactive substances used (74.7%). Most individuals would only tell the truth about their psychoactive substance use if they trust the doctor (53%). The details are shown in Table 5.

## 4. Discussion

This year’s PolDrugs survey is significant in two ways. First, it presents the latest demographic–epidemiological data on recreational users of psychoactive substances, which in itself is of value, as there are no similar studies on the Polish population. Moreover, the above data were collected from the same groups as the data collected in “PolDrugs 2021”, which allows for a fair comparison and evaluation of the data. However, they were collected over a longer period of time (four weeks instead of one week) and the number of responses collected was more than half the number collected in “PolDrugs 2021”. This can be explained by the fact that the data for the previous project were collected during the nationwide lockdown due to the COVID-19 pandemic, which might have led to greater activity by respondents on social media and, thus, provided more opportunity to view the survey. In addition, the fact that places associated with daily leisure activities (such as movie theaters, restaurants, and gyms) were closed may have led to more people being willing to participate in the survey.

The most surprising observation is the significant increase in the use of hallucinogenic mushrooms. In this year’s edition of PolDrugs, 57.6% of respondents reported having used hallucinogenic mushrooms in their lifetime and 42% in the past 12 months, while the figures from the 2021 edition were 42.7% and 28.9%, respectively. Thus, hallucinogenic mushrooms have overtaken amphetamines to become the third most commonly used stimulant after marijuana and MDMA, along with alcohol and nicotine. This is probably due to the increasing popularization of psychedelics in the media and the organization of various scientific and popular science conferences, including in Poland, where the first national conference on “Psychedelic Science” was held in September 2022, an interdisciplinary event organized by the Polish Psychedelic Society, the Polish Drug Policy Network, and the Faculty of Law and Administration of the University of Warsaw [6]. Media coverage on the topic is no accident, as scientific interest in the topic of psychedelics is noticeably increasing. For example, according to the PubMed Central database of medical publications from the U.S. National Institutes of Health’s National Library of Medicine, 141 publications were published on the keyword “psilocybin” in 2020, while in 2022 there were already 342 publications (data checked on 15 February 2023). Given the considerable public interest in the topic of psychedelics, as an increase in the use of DMT and a decrease in the use of LSD can be observed, the use of which remains objectively high anyway, it seems extremely important to consider comprehensive education in this area as one of the priorities in the field of public health, not only in Poland, as the topic is being covered by major international media [7,8,9]. In the survey, we asked about hallucinogenic mushrooms in the sense of psilocybin-containing mushrooms. According to the activists from the Social Drug Policy Initiative, it is reasonable to ask in the future not only about psilocybin-containing mushrooms, but also about other potentially hallucinogenic mushrooms, such as *Amanita muscaria*, since the activists have noticed an increase in the number of people who want to consume preparations of this type of mushrooms for recreational purposes in recent years. However, we could not find any reports on this phenomenon in the scientific literature, except for a brief report that shamans in eastern Siberia used this mushroom as an intoxicant and hallucinogen [10]. It is possible that this is a relatively new phenomenon that is just gaining momentum, and physicians may soon be confronted with its complications.

It was found that the surveyed users now most often earn more than PLN 5000, while previously the salary earnt was PLN 2000–3000. This fact can be explained by the unstable economic situation in Europe, the exchange rate of the Polish currency against the dollar and inflation, which gained momentum around the world during the COVID-19 pandemic and was accelerated by Russia’s attack on Ukraine, which led to some macroeconomic uncertainty around the world [11,12].

Interestingly, the proportion of people vaporizing tobacco has increased, while the proportion of people smoking classic cigarettes has decreased. In 2021, 37.4% of respondents resorted to classic cigarettes and 8.9% to vaporized tobacco, compared to 27.8% and 15.4% of respondents, respectively, in the current survey. It would be advisable to investigate this issue, because given the widespread marketing of such solutions in Polish stores where tobacco products can be purchased, it is doubtful that it is limited to this group of respondents. The COVID-19 pandemic may have affected the popularity of new forms of tobacco use. Many traditional cigarette stores closed or had limited hours, forcing many people to seek alternative sources of nicotine. In addition, many people who worked from home may have found themselves in a situation where they could consume heated tobacco products at home, which could have also increased their popularity. Finally, the pandemic may also have led to increased interest in health and ways to improve it, with some claiming that heated tobacco products are less harmful than conventional cigarettes, which is not clearly supported by scientific research. Little is known about the long-term health effects of tobacco vaporization. Jankowski et al. reported in their systematic review that more than half of the available data on the harms of tobacco vaporization systems come from the manufacturers, and Bravo-Gutiérrez et al. argued in their systematic review that the harms from using these devices include mechanisms that have already been reported for conventional cigarettes, as well as new mechanisms specific to these devices, concluding that these devices pose a significant public health problem and should be regulated or avoided [13,14]. Further research on heated tobacco products is important because the effects of their long-term consumption, including possible serious health risks, may still be unknown. In addition, it is important to understand the impact of their use on the younger generation, who are particularly at risk of developing a nicotine use disorder.

The results on respondents measuring the doses of their psychoactive substances before consumption or testing such substances in a laboratory or the use of colorimetric reagents seem to be comparable. The question is whether current harm reduction and drug education efforts are sufficient. The fewer the people that measure and test their substances before use, the greater the risk of accidental intoxication and the higher the social costs associated with sudden hospitalization or death. The construction of economic models that clearly describe this phenomenon could be an important tool in the discussion with decision-makers on the financing of preventive measures. This type of research should be conducted in a multidisciplinary manner, as economists have an important role to play.

The percentage of individuals most likely to use psychoactive substances in clubs and bars has increased slightly, and the percentage of those most likely to use these substances at music festivals has increased significantly (more than doubled). It is possible that this increase is due to the complete lifting of restrictions as a result of the COVID-19 pandemic. However, it is worth monitoring the situation over the next few years to see if these levels continue to change; education efforts may need to be adjusted by changing the target audiences. In addition, it is worthwhile studying the wellbeing of specific groups. Such multidirectional studies are regularly conducted and published, and should be continued in the future [15,16].

Overall, 58.3% of those surveyed this year have ever sought psychiatric treatment. In 2021, this figure was 31.9% of respondents, i.e., the percentage has almost doubled. This is alarming, but not surprising, as there is no doubt that the COVID-19 pandemic had a devastating impact on the mental health of the population [17]. The COVID-19 pandemic has affected many aspects of life, including people’s mental health. Psychoactive substance users are particularly vulnerable to emotional and psychological problems, which can be exacerbated by social isolation, limited access to medication, and difficulty in getting help. In addition, the pandemic has led to increased stress and uncertainty, which can increase the risk of addiction or lead to a relapse into mental illness for people with pre-existing health problems. All of these factors may contribute to an increase in the number of psychoactive substance users who need mental health care. In addition, there is currently a war going on at Poland’s eastern border, which is certainly not without impact on the mental wellbeing of the region’s inhabitants [18,19]. It seems important to monitor the state of mental health in the group of psychoactive substance users and conduct further research to determine the exact causes of such a phenomenon, taking into account as many factors as possible that could cause such a sharp increase.

## 5. Limitations

The manuscript is not free from limitations. The results presented here are from an online survey, which on the one hand allows respondents some freedom to express their opinions, but on the other hand does not preclude intentional bias in the survey. These results came from individuals who use social media extensively, and it is certain that there is a group of recreational users of psychoactive substances who do not use Facebook or Instagram, likely those in older age groups. Nonetheless, the fact that the results can be compared to those from two years ago, and the fact that social media is a very large part of daily life, encourages the publication of the above results.

## 6. Conclusions

1. The use of hallucinogenic mushrooms and DMT is increasing, which, combined with the widespread presence of the topic of psychedelics in the media, suggests that research and public education in this area should be among the priorities of public health efforts.
2. The percentage of people who vaporize tobacco is increasing, while the percentage of people who smoke traditional cigarettes is decreasing, which means that this phenomenon should be closely monitored for possible negative effects on human health.
3. In the group of psychoactive substance users, the percentage of people seeking psychiatric help has almost doubled in the last two years; this is an issue that needs to be studied in order to determine the causes of this phenomenon.

## Figures and Tables

**Table 1:** Detailed demographic data.

Variables		n	%
Age	13–17	34	3.0
	18–24	469	42.0
	25–30	321	28.8
	31–40	235	21.0
	41–62	58	5.2
Gender	Male	536	48.0
	Female	561	50.2
	Other	20	1.8
Sexual Orientation	Heterosexual	807	72.2
	Homosexual	65	5.8
	Bisexual	212	19.0
	Other	33	3.0
Relationship Status	Single	392	35.1
	In an informal relationship	546	48.9
	In a formal relationship	102	9.1
	In a polyamorous relationship	34	3.0
	Divorced	20	1.8
	Widower/widowed	2	0.2
	Other	21	1.9
Place of Residence	Large city (more than 200,000 inhabitants)	716	64.2
	Medium city (50,000 to 199,999 inhabitants)	180	16.1
	Small city (up to 49,999 inhabitants)	120	10.7
	Village	101	9.0
Education	I have a doctorate or higher degree	22	2.0
	Master’s degree or equivalent	277	24.8
	Bachelor’s degree or equivalent	245	21.9
	Medium	506	45.3
	Middle School	32	2.9
	Basic	35	3.1
Profession	Pupil: working	33	3.0
	Pupil: unemployed	62	5.6
	Student: working	202	18.1
	Student: unemployed	130	11.6
	Full-time employee	454	40.6
	Casual worker	35	3.1
	Retired or pensioner	4	0.4
	Own business	170	15.2
	Unemployed	27	2.4
Net Monthly Earnings (EUR 1 = approximately PLN 4.5)	Not applicable	220	19.7
	Up to PLN 1000	39	3.5
	PLN 1000–2000	82	7.3
	PLN 2000–3000	111	9.9
	PLN 3000–4000	174	15.6
	PLN 4000–5000	137	12.3
	Over PLN 5000	354	31.7

PLN: Polish zloty.

**Table 2:** Detailed results on nicotine and alcohol consumption.

Variables		n	%
Are you taking nicotine?	Yes, mostly classic cigarettes	311	27.8
	Yes, mostly tobacco vaporization (e.g., IQOS or the GLO system)	172	15.4
	Yes, mostly e-cigarettes (liquid)	157	14.1
	Yes, others	64	5.7
	No	413	37.0
Do you use alcohol?	Yes	907	81.2
	No	210	18.8
How often do you consume alcohol? (n = 907)	Once every few months or less frequently	70	7.7
	Once a month	107	11.8
	2–3 times a month	248	27.4
	4–5 times a month	199	21.9
	6–8 times a month	128	14.1
	9–10 times a month	58	6.4
	More than 10 times a month	97	10.7
What type of alcohol do you consume most often? (n = 907)	Beer	457	50.4
	Cocktails	112	12.3
	Wine	210	23.2
	Liqueurs, including flavored vodkas	25	2.8
	Vodka or other spirits	93	10.3
	Other	10	10.1
Have you ever thought about getting help because of your alcohol consumption? (n = 907)	Yes	117	12.9
	No	790	87.1

**Table 3:** Detailed results on psychoactive substance use.

Variables		n	%
Age of first exposure to psychoactive substances	10–12	38	3.4
	13–14	198	17.7
	15–17	499	44.7
	18–21	259	23.2
	22–26	88	7.9
	27–30	18	1.6
	31–40	11	1.0
	41–62	6	0.5
What substances have you used throughout your life?	Marijuana	1096	98.1
	MDMA/ecstasy	834	74.7
	Hallucinogenic mushrooms	643	57.6
	Amphetamine	641	57.4
	LSD or other lysergamides	590	52.8
	Mephedrone or other synthetic cathinones	536	48.0
	Cocaine	498	44.6
	Benzodiazepines	332	29.7
	Ketamine	262	23.5
	Other than those mentioned in the survey	208	18.6
	Opioids other than morphine, heroin, and fentanyl	185	16.6
	Methamphetamine	185	16.6
	DMT or plants containing DMT	169	15.1
	Synthetic cannabinoids	132	11.8
	Alkyl nitrites (“poppers”)	131	11.7
	Morphine	71	6.4
	Mescaline	31	2.8
	Benzofurans	23	2.1
	Heroin	21	1.9
	Fentanyl	18	1.6
	Aminorexes	3	0.3
What substances have you consumed in the past 12 months?	Marijuana	963	86.2
	MDMA/ecstasy	587	52.6
	Hallucinogenic mushrooms	469	42.0
	LSD or other lysergamides	360	32.2
	Amphetamine	356	31.9
	Mephedrone or other synthetic cathinones	343	30.7
	Cocaine	284	25.4
	Benzodiazepines	164	14.7
	Ketamine	126	11.3
	Other than those mentioned in the survey	96	8.6
	Methamphetamine	87	7.8
	DMT or plants containing DMT	75	6.7
	Opioids other than morphine, heroin, and fentanyl	73	6.5
	Alkyl nitrites (“poppers”)	45	4.0
	Morphine	21	1.9
	Synthetic cannabinoids	20	1.8
	Fentanyl	9	0.8
	Mescaline	8	0.7
	Benzofurans	7	0.6
	Heroin	6	0.5
	Aminorexes	0	0.0
How often do you use psychoactive substances other than alcohol?	Once every few months or less frequently	390	34.9
	Once a month	190	17.0
	2–3 times a month	158	14.2
	4–5 times a month	75	6.7
	6–8 times a month	56	5.0
	9–10 times a month	35	3.1
	More than 10 times a month	213	19.1
Who do you use substances with most often?	By yourself	225	20.2
	With a partner	251	22.5
	With family	10	0.9
	With friends	607	54.3
	Other than the above	24	2.1
Where do you use psychoactive substances most often?	At home	531	47.5
	At someone’s home	269	24.2
	In clubs	122	10.9
	In bars	8	0.7
	At music festivals	64	5.7
	Outdoors (forests, meadows)	123	11.0
Where do you buy your psychoactive substances most often?	From friends	641	57.4
	From a dealer or from strangers at music festivals/clubs or bars	220	19.7
	Via the darknet (Tor)	54	4.8
	Other options not included in the survey	202	18.1
Do you test your psychoactive substances with colorimetric reagents or send them to a lab for testing?	No, never	866	77.5
	Sometimes	205	18.4
	Yes, always	46	4.1
How much do you spend on psychoactive substances in a month?	Up to PLN 100	466	41.7
	PLN 100–200	165	14.8
	PLN 200–300	115	10.4
	PLN 300–400	83	7.4
	More than PLN 500	101	9.0
	I don’t spend money, I get it from someone	187	16.7
Do you measure the doses of your psychoactive substances?	Yes, always	424	38.0
	Sometimes	160	14.3
	Mostly by visual observation (“eyeballing”)	457	40.9
	Never	76	6.8
Do you educate yourself about the safety and use of psychoactive substances? (e.g., academic articles, NGOs, influencer channels)	Yes	1035	92.7
	No	82	7.3
Have you ever been in trouble with the law because of your use of psychoactive substances?	Yes	101	9.0
	No	1016	91.0
Have you ever neglected your daily responsibilities (work, study, family life) due to using psychoactive substances?	No, never	578	51.7
	Yes, once	105	9.4
	Yes, several times	378	33.9
	Yes, it happens to me often	56	5.0

**Table 4:** Detailed results on substance use related professional help.

Variable		n	%
Have you thought about seeking professional help for your psychoactive substance use?	Yes	195	17.5
	No	922	82.5
Have you ever sought medical help shortly after taking psychoactive substances? (e.g., for acute poisoning)	Yes	72	6.4
	No	1045	93.6
After taking which substance did you seek medical help?	Amphetamine	17	23.6
	Mephedrone or other synthetic cathinones	14	19.4
	I don’t know what substance I took	12	16.7
	Marijuana	12	16.7
	Opioids other than morphine, heroin, and fentanyl	9	12.5
	MDMA/ecstasy	8	11.1
	Benzodiazepines	8	11.1
	LSD or other lysergamides	6	8.3
	Cocaine	6	8.3
	Heroin	3	4.2
	Fentanyl	3	4.2
	Morphine	2	2.8
	Synthetic cannabinoids	2	2.8
	Hallucinogenic mushrooms	2	2.8
	Methamphetamine	1	1.4
	Ketamine	1	1.4
	Alkyl nitrites (“poppers”)	1	1.4

**Table 5:** Detailed results on psychiatric treatment.

Variable		n	%
Have you received psychiatric treatment?	Yes	466	41.7
	No	651	58.3
For what reason? For different diagnoses, please mark the first one that was made. (n = 466)	Depressive disorders	239	51.3
	Anxiety disorders	83	17.9
	ADHD	30	6.4
	Personality disorders	29	6.2
	Other	25	5.4
	Bipolar affective disorder	22	4.7
	Addiction	15	3.2
	Insomnia	10	2.1
	Adaptive disorders	9	1.9
	Schizophrenia	4	0.9
Where did the treatment take place? (n = 466)	Private practice	344	73.8
	Mental health clinic	160	34.3
	Addiction treatment clinic	37	7.9
	Psychiatric ward closed	60	12.9
	Psychiatric day ward	33	7.1
	Other	16	3.4
Have you ever made any suicide attempts? (n = 466)	Yes, once	82	17.6
	Yes, several times	89	19.1
	No	295	63.3
Did your use of psychoactive substances precede your first visit to a psychiatrist? (n = 466)	Yes	295	63.3
	No	171	36.7
Did the psychiatrist ask about the psychoactive substances used during the interview? (n = 466)	Yes	348	74.7
	No	118	25.3
When going to a psychiatrist, would you tell the truth about the psychoactive substances used?	Yes, always	383	34.3
	Rather yes, if the doctor inspired my confidence	592	53.0
	No, I’m afraid of the legal consequences	40	3.6
	No, I’m afraid of being judged	36	3.2
	No, I don’t want to have anything “in the papers”	41	3.7
	No, for other reasons	25	2.2

## Data Availability

Data available on reasonable request.
